# Enhanced production of Ca^2+^-polymalate (PMA) with high molecular mass by *Aureobasidium pullulans* var. *pullulans* MCW

**DOI:** 10.1186/s12934-015-0296-3

**Published:** 2015-08-07

**Authors:** Yu-Kuang Wang, Zhe Chi, Hai-Xiang Zhou, Guang-Lei Liu, Zhen-Ming Chi

**Affiliations:** College of Marine Life Sciences, Ocean University of China, Yushan Road, No. 5, Qingdao, 266003 China

**Keywords:** Polymalate, *Aureobasidium pullulans* var. *pullulans*, Corn steep liquor, Fermentation, High molecular weight of PMA

## Abstract

**Background:**

Polymalic acid (PMA) has many applications in food and medical industries. However, so far it has not been commercially produced by fermentation. Therefore, it is very important how to develop an economical process for a large scale production of PMA by one step fermentation.

**Results:**

After over 200 strains of *Aureobasidium* spp. isolated from the mangrove systems in the South of China were screened for their ability to produce Ca^2+^-polymalate (PMA), it was found that *Aureobasidium pullulans* var. *pullulans* MCW strain among them could produce high level of Ca^2+^-PMA. The medium containing only 140.0 g/L glucose, 65.0 g/L CaCO_3_ and 7.5 g/L corn steep liquor was found to be the most suitable for Ca^2+^-PMA production. Then, 121.3 g/L of Ca^2+^-PMA was produced by *A. pullulans* var. *pullulans* MCW strain within 120 h at flask level. During 10-L batch fermentation, 152.52 g/L of Ca^2+^-PMA in the culture and 8.6 g/L of cell dry weight were obtained within 96 h, leaving 4.5 g/L of reducing sugar in the fermented medium. After purification of the Ca^2+^-PMA from the culture and acid hydrolysis of the purified Ca^2+^-PMA, HPLC analysis showed that *A.pullulans* var. *pullulans* MCW strain produced only one main component of Ca^2+^-PMA and the hydrolysate of the purified Ca^2+^-PMA was mainly composed of l-malic acid. Mw (the apparent molecular weight) of the purified PMA was 2.054 × 10^5^ (g/moL) and the purified PMA was estimated to be composed of 1784 l-malic acids.

**Conclusions:**

It was found that *A. pullulans* var. *pullulans* MCW strain obtained in this study could yield 152.52 g/L of Ca^2+^-PMA within the short time, the produced PMA had the highest molecular weight and the medium for production of Ca^2+^- PMA by this yeast was very simple.

## Background

Polymalate (PMA) which is a linear anionic polyester consisting of monomeric repeating units of l-malate has received a significant attention because it can be used as a pro-drug or in a drug-delivery system [1]. PMA can be easily dissolved in water and degraded quickly in liquid. It is a biodegradable, biocompatible, non-immunological, bioabsorbable and nontoxic polyester. Its hydrolysate, l-malic acid, can be metabolized in the tricarboxylic acid cycle in any organisms, offering energy and carbon skeleton [2]. Carboxyl group present in the side chain of PMA can readily react with other functional groups, such as carboxy, hydroxyl and amino. Therefore, the PMA-based nanoconjugates and nanoparticles consisting of several functional modules such as antibodies and drugs have been recently used for a specific cancer chemotherapy in human and animals [3]. In this case, it is necessary to develop an economical process for a large scale of PMA production by one step fermentation. PMA can be chemically synthesized by two approaches: i.e. ring-opening polymerization and direct polycondensation at high temperature (over 110–140°C). However, the processes are too complex, the reaction conditions are not environment-friendly and its raw material is a petroleum derivative, maleic anhydride [4]. Indeed, PMA is being produced from renewable sugars such as glucose by one step fermentation. Although *Penicillium cyclopium* and *Physarum polycephalum* [5, 6] could produce PMA, the titer of PMA (2.7 g/L) produced by *P. polycephalum* is too low to use it for a large-scale of PMA production by fermentation [7]. In recent years, it has been found that a large quantity of PMA can be produced by different strains of *Aureobasdium* spp. isolated from different environments [8–10]. Some strains of *Aureobasdium* spp. isolated from the phylloplane and fresh plant samples could produce around 60.0 g/L of PMA [9–12]. However, so far it has not been commercially produced by fermentation [13].

In our previous studies [13], it was found that many strains of *Aureobasidium* spp. isolated from the mangrove ecosystems could produce high level of Ca^2+^-PMA. For example, 118.3 g/L of Ca^2+^-PMA in the culture and 16.4 g/L of cell dry weight were yielded by a novel *Aureobasidium* sp. P6 strain within 168 h. In this present study, we found that another yeast strain MCW isolated from the same mangrove ecosystem also could produce high level of Ca^2+^-PMA in a simple medium. It has been evidenced that biotin-dependant pyruvate carboxylase can play an important role in biosynthesis of l-malate and other C4 dicarboxylic acids [14] and corn steep liquor (CSL) can stimulate l-malate production by *Penicillium viticola* 152 isolated from marine algae [15]. Therefore, effects of corn steep liquor on Ca^2+^-PMA production by the yeast strain MCW were also examined in this study.

## Results and discussion

### Characterization of the yeast strain MCW

In our previous study [13], after the ability to yield Ca^2+^-PMA by over 200 strains of *Aureobasidium* spp. isolated from the mangrove systems in Hainan, China, was examined, it was found that the yeast strain P6 which was identified to be one novel yeast strain of *Aureobasidium* spp. could produce the high level of Ca^2+^-PMA (90.0 g/L). At the same time, we found that another yeast strain MCW also could produce more than 90.0 g/L of Ca^2+^-PMA (data not shown). Our results showed that the morphologies of the colonies and the cells of the yeast strain MCW were obviously different from those of the colonies and the cells of *Aureobasidium* sp. P6 [13] (data not shown). However, the morphologies of the colonies and the cells of the yeast strain MCW were similar to those of the colonies and the cells of *Aureobasidium pullulans* var. *pullulans* [16].

**Fig. 1:**
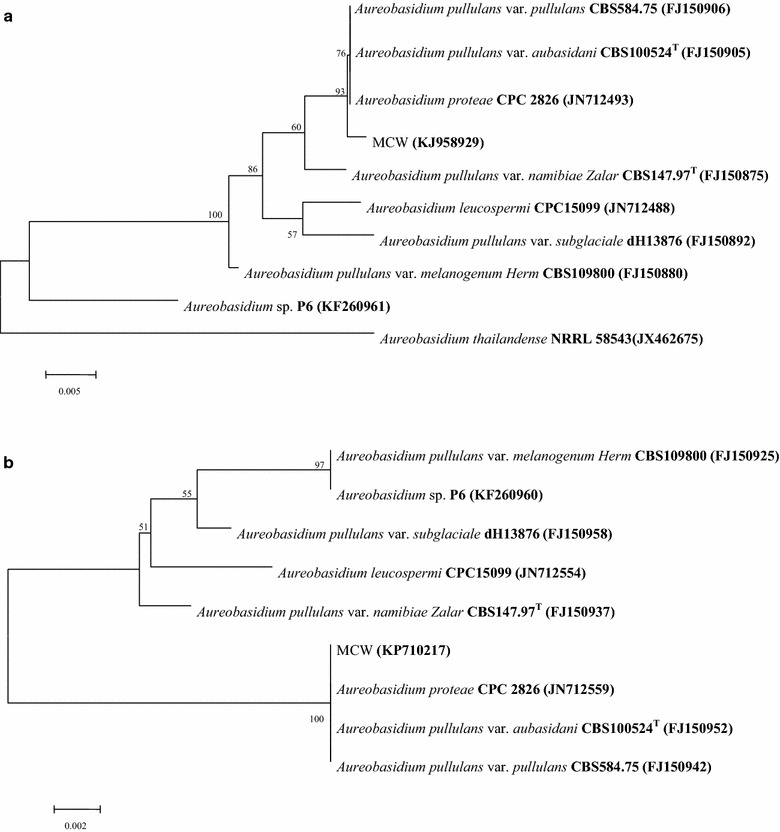
The phylogenetic trees of the yeast strain MCW and other yeast relatives based on a neighbor-joining analysis of ITS sequences (**a**) and 26S rDNA sequences (**b**). Bootstrap values (1,000 pseudoreplications) were ≥52%.

After the fermentation tests and carbon source assimilation experiments using the yeast strain MCW were performed [22], our results also showed that the characteristics of the yeast strain MCW were closely related to those of the typical strain *A. pullulans* var. *pullulans* CBS 584.75 (data not shown). After ITS sequence (the accession number was KJ958929) and 26S rDNA sequence (the accession number was KP710217) of the yeast strain MCW were PCR amplified, determined and aligned, the phylogenetic trees were constructed as described in “Methods”. Both the analysis for the similarity between ITS of the yeast strain MCW and that in the NCBI database and the analysis for similarity between 26S rDNA sequence of the yeast strain MCW and that in the NCBI database indicated that many phylogenetically related yeast species were similar to the yeast strain MCW used in this study (Fig. 1). The topology of the phylograms in Fig. 1a confirmed that the yeast strain MCW was identified to be one strain of *A.pullulans* var. *pullulans* or *A.pullulans* var. *aubasidani* or *A. proteae* because the similarity between ITS of the yeast strain MCW and that of the type strain *A. pullulans* var. *pullulans* CBS 584.75 or that of *A. pullulans* var. *pullulans* CBS100524^T^ or that of *A.pullulans* var. *aubasidani* CBS100524^T^ or *A. proteae* CPC 2826 were the same (99.79%) (Table 1). However, the topology of the phylograms in Fig. 1b confirmed that the yeast strain MCW was identified to be one strain of *A.pullulans* var. *pullulans* because the similarity between 26S rDNA sequence of the yeast strain MCW and that of the type strain *A. pullulans* var. *pullulans* CBS 584.75 was the highest (99.64%) (Table 1). So far, most of the yeast strains which can produce a large amount of PMA have been thought to be *A. pullulans* [9–11, 17, 18]. In fact, *A. pullulans* can be divided into five varieties: *A. pullulans* var. *pullulans*, *A. pullulans* var. *aubasidani, A. pullulans* var. *namibiae, A. pullulans* var. *melanogenum* and *A. pullulans* var. *subglaciale* [2, 19]. However, it is not clear to which variety most of the yeast strains available which can produce a large amount of PMA belong and the taxonomic positions of the high PMA producers of the *A. pullulans* are still unknown [2]. In our previous study [13], *Aureobasidium* sp. P6 was also found to be able to produce a large amount of Ca^2+^-PMA. It has been evidenced that different strains of *A. pullulans* and *Aureobasidium* sp. P6 can accumulate more than 50 g of Ca^2+^-PMA per liter of the broth [10, 11, 13]. In contrast, the results in Fig. 1b showed that *A. pullulans* var. *pullulans* MCW strain isolated from the mangrove ecosystem also could produce high level of Ca^2+^-PMA. Therefore, *A. pullulans* var. *pullulans* MCW isolated from the mangrove ecosystem was a new producer of Ca^2+^-PMA and could be a candidate for high level of Ca^2+^-PMA production. So it was used in the subsequent investigations.

**Table 1:** Similarity between ITSs or between 26S rDNAs of the yeast strain MCW and other strains of *Aureobasidium* spp

Similarity of ITS (%)	The relatives
99.79	*A. pullulans* var. *aubasidani* CBS100524^T^
98.94	*A. pullulans* var. *namibiae* Zalar CBS147.97^T^
99.79	*A. proteae* CPC 2826
98.14	*A. leucospermi* CPC15099
98.56	*A. pullulans* var. *melanogenum* Herm CBS109800
99.79	*A. pullulans* var. *pullulans* CBS584.75
96.89	*A. pullulans* var. *subglaciale* dH13876
99.79	*A. pullulans* var. *pullulans* CBS100524^T^
93.74	*Aureobasidium* sp. P6
92.70	*A. thailandense* NRRL 58543

Similarity of 26S rDNA (%)	The relatives
99.46	*A. pullulans* var. *aubasidani* CBS100524^T^
97.47	*A. pullulans* var. *namibiae* Zalar CBS147.97^T^
98.78	*A. proteae* CPC 2826
96.34	*A. leucospermi* CPC15099
96.93	*A. pullulans* var. *melanogenum* Herm CBS109800
99.64	*A. pullulans* var. *pullulans* CBS584.75
97.29	*A. pullulans* var. *subglaciale* dH13876
99.46	*A. pullulans* var. *pullulans* CBS100524^T^
96.49	*Aureobasidium* sp. P6

The accession number of ITS of the yeast strain MCW was KJ958929, and the accession number of 26S rDNA of the yeast strain MCW was KP710217.

### Effects of different concentrations of glucose and CaCO_3_ on Ca^2+^-PMA production

**Fig. 2:**
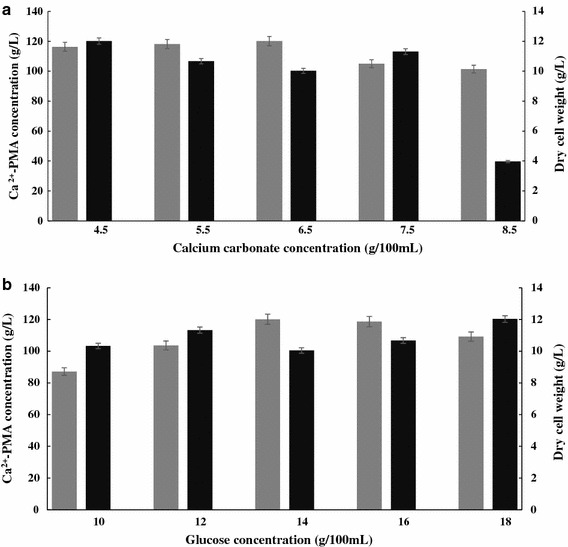
Effects of different concentrations of CaCO_3_ (**a**) and glucose (**b**) on Ca^2**+**^ **-**PMA production (*grey*) and cell growth (*black*) by the yeast strain MCW. Data are given as mean ± SD, n = 3.

It has been well documented that a high initial C/N ratio (nitrogen starvation) and the presence of CaCO_3_ in the medium are required to boost l-malic acid biosynthesis for PMA production in microbial cells [11, 14]. Therefore, it is very important to optimize the glucose and CaCO_3_ concentrations in the Ca^2+^-PMA production medium. So the effects of different concentrations of glucose and CaCO_3_ on Ca^2+^-PMA production and cell growth by the yeast strain MCW were examined as described in “Methods”. The results in Fig. 2b showed that when the Ca^2+^-PMA production medium contained 140.0 g/L glucose, the amount of the Ca^2+^-PMA in the culture reached the highest (121.11 g/L) while the results in Fig. 2a showed that when the Ca^2+^-PMA production medium contained 65.0 g/L of CaCO_3_, the amount of the Ca^2+^-PMA in the culture was the highest (121.13 g/L). The results in Fig. 2 also revealed that the Ca^2+^-PMA production by the yeast strain MCW was closely related to its cell growth. The data of the Ca^2+^-PMA titers and cell mass obtained above were subjected to One-way Analysis of Variance (ANOVA) [20]. *P* values were calculated by Student’s *t* test (n = 3). *P* values less than 0.05 were considered statistically significant. The statistical analysis was performed using SPSS 11.5 for Windows (SPSS Inc., Chicago, IL, USA). The results demonstrated that there were big differences between the Ca^2+^-PMA titers and cell mass shown in Fig. 2. It has been reported that during the nitrogen starvation (a high initial C/N ratio in the production medium), expression of most of the glycolytic genes and all the genes related to the cytosolic l-malic acid production pathway in *Aspergillus oryzae* were highly upregulated [21]. It also has been evidenced that CaCO_3_ is necessary for l-malate production in the fermentation medium because the presence of CaCO_3_ in the medium can keep pH constant of around 6.5 and provide CO_2_ as a substrate for efficient production of l-malate, the precursor of PMA biosynthesis [14, 22]. The results in Fig. 2 were indeed consistent the finding. It was thought that in the nitrogen starvation, many zinc finger proteins such as Msn2/4, Gat1, Gln3 and AreA are involved in biosynthesis of organic acids, single cell oils, exopolysaccharides, antibiotics, toxins and others in fungal cells [23, 24, 28]. However, it is still completely unknown if such zinc finger proteins could be involved in PMA biosynthesis in *A. pullulans* var. *pullulans* MCW strain used in this study. Such investigation is being carried out in this laboratory.

### Effects of different concentrations of corn steep liquor (CSL) on Ca^2+^-PMA production

**Fig. 3:**
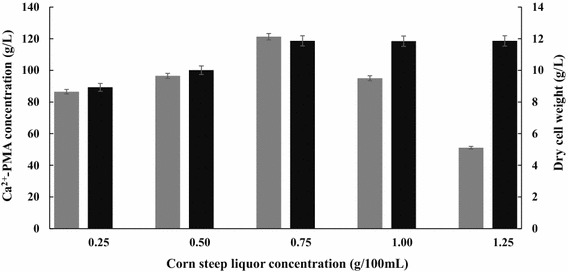
Effects of different concentrations of corn steep liquor on Ca^2+^-PMA production (*grey*) and cell growth (*black*) by the yeast strain MCW. Data are given as mean ± SD, n = 3.

**Fig. 4:**
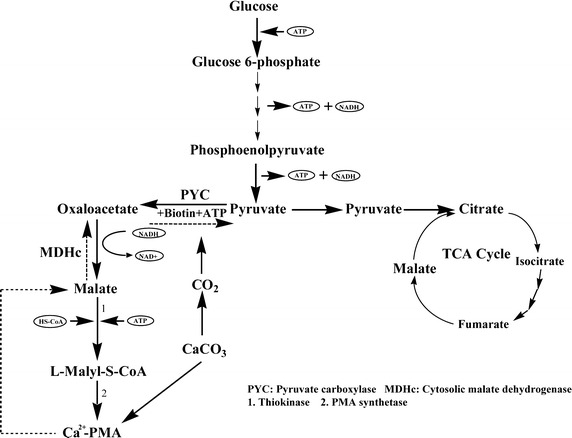
The proposed pathway of the PMA metabolism in the yeast cells.

CSL has been reported to have a rich source of nitrogen, water soluble vitamins including biotin, amino acids, minerals and other stimulants [25]. Therefore, effects of different concentrations of CSL on Ca^2+^-PMA production and cell growth by the yeast strain MCW were tested. It can be observed from the data in Fig. 3 that when the initial CSL concentrations in the PMA production medium were increased from 2.5 to 7.5 g/L, the Ca^2+^-PMA titers were also significantly increased from 86.54 to 121.31 g/L. However, when the initial CSL concentrations in the PMA production medium were increased from 7.5 to 12.5 g/L, the Ca^2+^-PMA titers were rapidly decreased from 121.31 to 51.12 g/L (Fig. 3). Therefore, 7.5 g/L of CSL in the PMA production medium was the most suitable for Ca^2+^-PMA production by the yeast strain MCW. This meant that like the calcium malate production [15], CSL indeed greatly promoted the Ca^2+^-PMA production by the yeast strain MCW. Furthermore, addition of CSL could significantly simplify the Ca^2+^-PMA production medium because the medium only contained glucose, CaCO_3_ and CSL. However, as the initial CSL concentrations in the Ca^2+^-PMA production medium were increased from 2.5 to 12.5 g/L, its cell growth was also increased continuously (Fig. 3). The data of the Ca^2+^-PMA titers and cell mass obtained above were subjected to One-way Analysis of Variance (ANOVA) [20]. *P* values were calculated by Student’s *t* test (n = 3). *P* values less than 0.05 were considered statistically significant. The statistical analysis was performed using SPSS 11.5 for Windows (SPSS Inc., Chicago, IL, USA). The results demonstrated that there were big differences between the Ca^2+^-PMA titers and cell mass shown in Fig. 3. According to the pathway of PMA biosynthesis [2], pyruvate carboxylase (PYC) and malate dehydrogenase (MDH) are involved in PMA biosynthesis in the non-oxidative pathway (Fig. 4). It has been known that PYC is a biotin-dependent tetrameric enzyme that catalyzes the carboxylation of pyruvic acid to form oxaloacetic acid, suggesting that biotin is required during biosynthesis of PMA [26]. Therefore, in the presence of CSL, activities of PYC and MDH may be promoted so that l-malic acid biosynthesis was enhanced, causing high level production of PMA by the yeast strain MCW (Fig. 3). In addition, CSL was also found to stimulate malate production by *P. viticola* 152 isolated from marine algae [15]. However, the exact mechanisms of the promotion of the activities of PYC and MDH and the Ca^2+^-PMA production by CSL are still completely unknown. Maybe biotin present in CSL was required by PYC.

### Ca^2+^-PMA production by 10-L fermentation

**Fig. 5:**
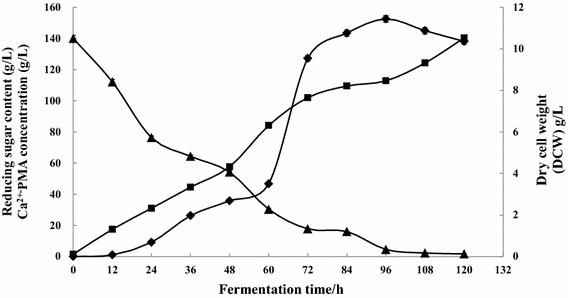
The time course of Ca^2+^-PMA production (*filled diamond*), cell growth (*filled square*) and the changes in the amount of reducing sugar (*filled triangle*) during the 10-L fermentation. Data are given as mean ± SD, n = 3.

In order to know if the Ca^2+^-PMA production from glucose and CaCO_3_ by one step fermentation can be repeated in the fermentor, the 10-L fermentation was carried out as described in “Methods”. During the 10-L fermentation, the changes in Ca^2+^-PMA titer, cell mass, and reducing sugar concentration were monitored. The results in Fig. 5 showed that during the 10-L fermentation, 152.52 g/L of Ca^2+^-PMA in the fermented medium was achieved from 140.0 g/L glucose, 65.0 g/L CaCO_3_ and 7.5 g/L CSL and the biomass in the culture was 8.59 g/L within 96 h, leaving 4.5 g/l of reducing sugar in the fermented medium. It also can be observed from the data in Fig. 5 that a Ca^2+^-PMA yield of 1.13 g/g of glucose, a volumetric Ca^2+^-PMA productivity of 1.59 g/L/h and a specific Ca^2+^-PMA productivity of 0.012 g/g/h were reached within 96 h of the fermentation, demonstrating that the titer, yield, and productivity of the Ca^2+^-PMA by this yeast strain MCW were very high and the fermentation period was very short. However, after 96 h of the fermentation, the titer of Ca^2+^-PMA was decreased and cell growth was still continuously increased (Fig. 5). This may be due to that the Ca^2+^-PMA was degraded after 96 h of the fermentation and the produced malate was used for cell growth according to the metabolism pathway of PMA in Fig. 4. In our previous study [13], 118.3 g/L of Ca^2+^-PMA was yielded by *Aureobasidium* sp. P6 within 168 h, the volumetric productivity was 0.67 g/L/h and the yield was 0.87 g/g [13]. In the 10-L fermentor, *A. pullulans* ZD-3d isolated from the terrestrial source produced a high PMA concentration (57.2 g/L) and a volumetric productivity (0.35/L/h) was achieved within 160 h when the fermentation medium contained 120.0 g/L of glucose and 30.0 g/L of CaCO_3_ [11]. In another study [9], *A. pullulans* ipe-1 could produce 37.9 g/L of PMA and a yield of 0.3 g/g was reached. Around 63.2 g/L of PMA with a volumetric productivity of 1.15 g/L/h was obtained by the same yeast strain during the repeated-batch cultivation [9]. When *A. pullulans* CBS 591.75 was grown in the stirred-tank reactor, it could produce 9.8 g/L of PMA within 9 days and a yield of 0.11 g/g was got [18]. *A. pullulans* strain ZX-10 grown in the medium with 120.0 g/L of glucose produced 50.0 g/L of PMA during the batch fermentation and the high productivity was 0.61 g/L/h in a free-cell fermentation in a stirred-tank bioreactor [10]. This demonstrated that *A. pullulans* var. *pullulans* MCW strain used in this study may be the most suitable yeast strain for Ca^2+^-PMA production from glucose on a large scale in industry because it could produce much more Ca^2+^-PMA than any other yeast strains reported so far and the production medium was very simple (Figs. 2, 3 and 5).

### Analysis of the fermentation products and hydrolysate of the fermentation products by HPLC

**Fig. 6:**
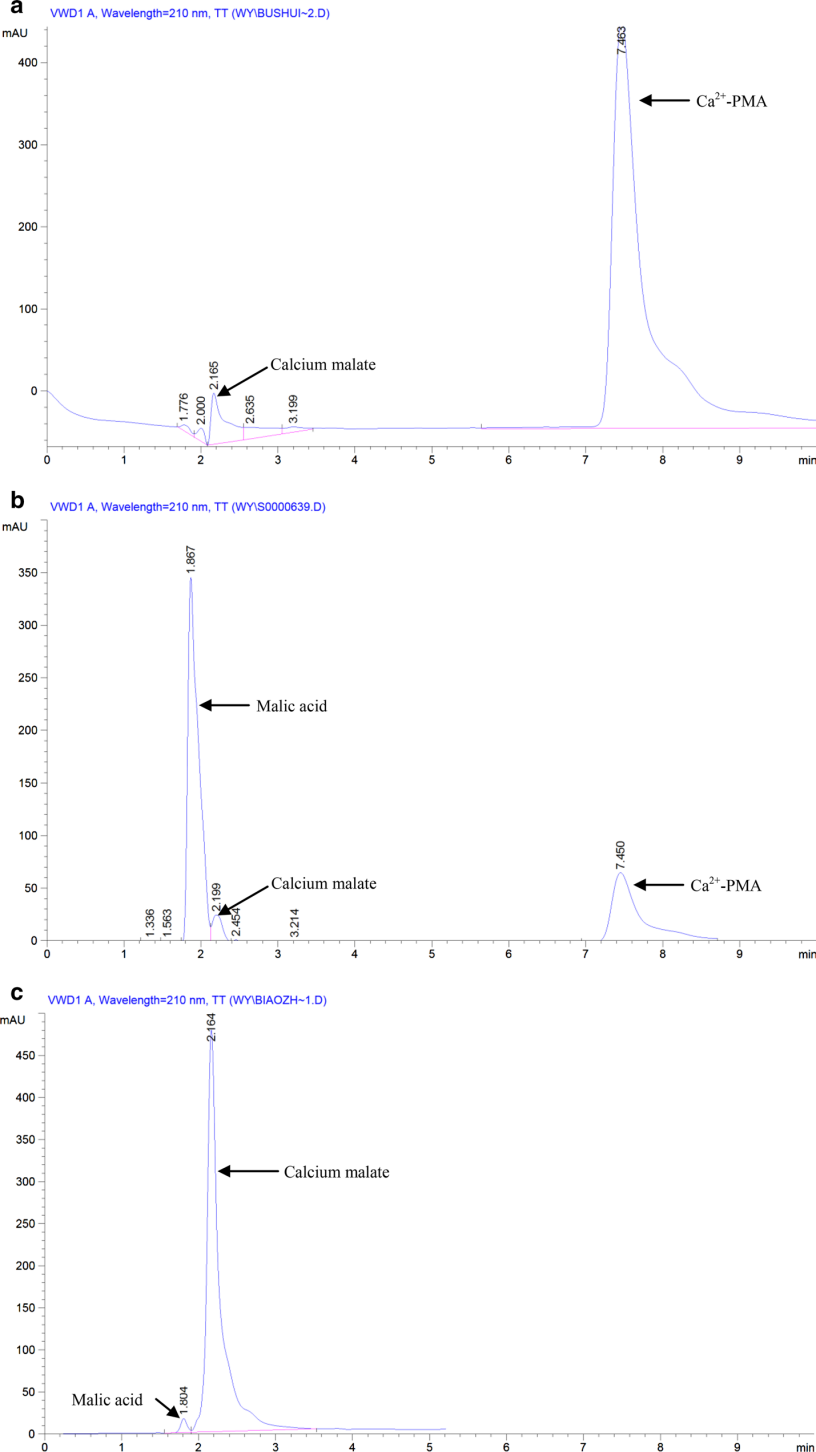
HPLC chromatogram of standard calcium malate and malic acid (**c**), fermentation product (**a**), and hydrolysate of the fermentation product using H_2_SO_4_ as the acid (**b**).

After the analysis of the purified precipitate with HPLC, the results in Fig. 6a confirmed that the major fermentation product was composed of one component and the minor component was calcium malate, the precursor of PMA, indicating that the main product produced by the yeast strain MCW was Ca^2+^-PMA. It also can be seen from the results in Fig. 6b that after acid hydrolysis of the fermentation product, the hydrolysates were composed of major l-malic acid and minor calcium malate and Ca^2+^-PMA. In contrast, the yeast strain P6 produced two components of Ca^2+^-PMA, and the hydrolysate of the Ca^2+^-PMA only contained calcium malate [13]. However, after the acid hydrolysis of the fermentation products produced by *A. pullulans* strain ZX-10, the hydrolysates contained acetic acid and malic acid [10]. This again confirmed that *A. pullulans* var. *pullulans* MCW strain used in this study may be the most suitable yeast strain for Ca^2+^-PMA production from glucose on a large scale in industry.

### Molecular mass of the purified PMA

After the measurement of the molecular mass of the purified PMA by the GPC, the results in Table 2 showed that Mn, Mw (the apparent molecular weight) and Mz were 1.627 × 10^5^ (g/moL), 2.054 × 10^5^ (g/moL) and 3.091 × 10^5^ (g/moL), respectively. According to the molecular mass of the monomeric repeating unit (l-malic acid) of the PMA molecule, each of the PMA molecule produced by *A. pullulans* var. *pullulans* MCW strain was estimated to be composed of 1,784 l-malic acids. The results in Table 2 also showed that the polydispersity (Mw/Mn) of the purified PMA was 1.263 (1.0%), suggesting that the produced PMA had narrow molecular mass distribution. Indeed, the peak limits of the purified PMA were in the range of 26.405–33.916 min (Table 2). The apparent molecular masses of the PMA produced by different strains of *A.pullulans* were estimated to be distributed between about 3,000 and 11,000 g/moL [11, 12, 18, 27, 28] while the Mn and polydispersity of the PMA produced by *A. pullulans* ZD-3d were 4,247 and 1.09, respectively [11]. In contrast, the relative molecular mass of the PMA produced by *Physarumpolycephalum* had an average mass of 50,000 g/mol (polydispersity of 2.0) [8]. This meant that the molecular mass of the PMA produced by *A. pullulans* var. *pullulans* MCW strain used in this study was much higher than that of the PMAs produced by any other strains. We think that the PMA with high molecular mass may be a good candidate in making biomaterials because PMA is regarded as a promising building block for the design of efficient drug delivery systems of the nanoconjugate and nanoparticles [3]. The higher the molecular mass of PMA, the higher the strength of the nanoconjugate and nanoparticles based on PMA [3, 4].

**Table 2:** Analysis of the purified PMA using a gel permeation chromatography (GPC)

Items	Results
Peak limits (min)	26.405–33.916
Mn (g/moL)	1.627e+5 (0.7%)
Mw (g/moL)	2.054e+5 (0.7%)
Mp (g/moL)	1.587e+5 (0.4%)
Mz (g/moL)	3.091e+5 (2%)
Mz/Mn	1.900 (2%)
Mw/Mn	1.263 (1.0%)

*Mn* number-average molecular weight, *Mp* peak-position molecular weight, *Mz* z-avearge molecular weight

## Conclusions

*A. pullulans* var. *pullulans* MCW strain isolated from mangrove ecosystem could produce 152.52 g/L of Ca^2+^-PMA in the culture and 8.6 g/L of cell dry weight within 96 h in the presences of CSL. The purified Ca^2+^-PMA contained only one main component and the hydrolysate of Ca^2+^-PMA was mainly composed of malic acid. Mw of the purified PMA was 2.054 × 10^5^ (g/moL) and the purified PMA was composed of 1784 l-malic acids. This is the highest molecular mass of PMA obtained so far.

## Methods

### Yeast strain and media

The yeast strain MCW (collection number 2E01289 at the Marine Microorganisms Culture Collection of China-MCCC) of *Aureobasidium* spp. was isolated from the mangrove systems in Hainan Province of China and used in this study [13]. The Latitude and longitude of the sampling site are N19°53′ E110°19′. The medium for growth of the seed culture contained 60.0 g/L of glucose, 3.0 g/L of yeast extract, 3.0 g/L of NH_4_NO_3_, and 10.0 g/L of CaCO_3_. The cultivation time and temperature were 48 h and 28°C, respectively. The medium for Ca^2+^-PMA production consisted of 140.0 g/L of glucose, 7.5 g/L of corn steep liquor (CSL) and 65.0 g/L of CaCO_3_. The CSL was purchased from the local Corn Starch Company in Qingdao, China. The Potato-Dextrose-Agar (PDA) medium was 100 mL of potato extract containing 2.0 g glucose and 2.0 g agar.

### Morphological analysis

The yeast strain MCW isolated from the mangrove system was grown on PDA plate at 28°C for 6 days. Its colony and cell morphologies were observed and photographed employing a Olympus U-LH100HG fluorescent microscope with ×40 objective under blue light. The images of the cells were recorded using the cellSens Standard software.

### DNA extraction, PCR amplification, DNA sequencing, and phylogenetic analysis

The total genomic DNA from the marine yeast strain MCW was extracted according to the methods described by Sambrook et al. [29]. To evaluate phylogenetic relationships among the yeast strain MCW and the typical strains reported on internet, amplification and sequencing of ITS (Internal Transcribed Spacer) and 26S rDNA from the marine yeast strain MCW were performed using the primers IT5: 5′-TCCGTAGGTGAACCTGCGG-3′ and IT6: 5′-TCCTCCGCTTATTGATATGC-3′ [30] and the primers NL-1: (5′-GCATATCAATAAGCGGAGGAAAAG-3′) and NL-4: (5′-GGTCCGTGTTTCAAGACGG-3′) [31]. The sequences of ITS (the accession number of ITS was KJ958929) and 26S rDNA (the accession number of 26S rDNA was KP710217) obtained above were aligned using BLAST analysis (http://blast.ncbi.nlm.nih.gov/Blast.cgi, last accessed 18 October 2007). The sequences which shared over 98% similarity with currently available sequences on internet were considered to be the same species or the same varieties. The multiple alignments were conducted using Clustal × 1.83, and the phylogenetic trees were made using MEGA 4.0 [32].

### Optimization of the medium for Ca^2+^-PMA production

The yeast strain MCW was cultivated aerobically in the seed culture medium at 28°C and 180 rpm for 48 h. A total of 2.5 mL of the cultures was inoculated into the 250-mL flask containing 50.0 mL of the Ca^2+^-PMA production medium supplemented with different concentrations of glucose, corn steep liquor and CaCO_3._ The flasks were aerobically incubated at 28°C and 180 rpm for 5 days. The culture obtained was centrifuged at 6,000×*g* and 2°C for 10 min. Ca^2+^-PMA in the supernatant was obtained, and quantitative determination of Ca^2+^-PMA was performed as described below.

### Batch fermentation

Ca^2+^-PMA production by the yeast strain MCW was also performed in the 10-L fermenter. The seed culture was prepared as described above. The fermentation was carried out in a fermentor [BIOQ-6005-6010B, Huihetang Bio-Engineering Equipment (Shanghai) CO-LTD] equipped with baffles, a stirrer, a heating element, an oxygen sensor, and a temperature sensor. Three hundred milliliters of the seed culture were transferred into 6 L of the Ca^2+^-PMA production medium containing only 140.0 g/L of glucose, 7.5 g/L of CSL and 65.0 g/L of CaCO_3_. The fermentation was performed under the conditions of the agitation speed of 300 rpm, the aeration rate of 8 L/min, the temperature of 28°C, and the fermentation period of 120 h. During the fermentation, 10.0 mL of the culture was collected in the interval of 12 h and was centrifuged at 6,000×*g* and 2°C for 10 min, and Ca^2+^-PMA and reducing sugar (glucose) in the supernatant obtained were determined as described below. The cell dry weight in 10.0 mL of the culture during the 10-L fermentation was also measured as described below.

### Purification and calculation of the amount of the pure Ca^2+^-PMA

The culture obtained during the 10-L fermentation was centrifuged at 6,000×g and 2°C for 10 min. Ca^2+^-PMA in 10 mL of the supernatant was obtained with the repeated methanol precipitation. Briefly, the first addition of 5.0 mL of methanol was to selectively remove exopolysaccharide (EPS) as a precipitate. After the EPS was removed by centrifugation, 30.0 mL of methanol was added into the supernatant, and the mixture was incubated at 4°C for 12 h. The resulting Ca^2+^-PMA precipitates were collected by centrifugation at 6,000×*g* and 2°C for 10 min and dried by evaporating at 50°C. After the dried materials were re-dissolved in 5.0 mL of distilled water, 15.0 mL of methanol was added into the solution, and the mixture was incubated at 4°C for 12 h; the above procedures were repeated several times until the pure Ca^2+^-PMA was obtained. Finally, the amount of the purified Ca^2+^-PMA from 10 mL of the supernatant was weighed and the amount of Ca^2+^-PMA in 1.0 L of the culture was calculated.

### Determination of reducing sugar concentration in the culture

The amount of the reducing sugar in the culture was determined according to the Nelson–Somogyi method [33].

### Measurement of cell mass

The dried biomass in the culture was measured according to the methods described by Chi et al. [34].

### Acid hydrolysis of the purified Ca^2+^-PMA

The chemical degradation of the purified Ca^2+^-PMA was carried out with 0.5 M sulfuric acid in sealed glass tubes at 90°C. Calcium malate and l-malic acid formed during the hydrolysis of the purified Ca^2+^-PMA were assayed using HPLC as described below.

### Analysis of the purified Ca^2+^-PMAs and hydrolysate of them using HPLC

The purified Ca^2+^-PMA and hydrolysate of the purified Ca^2+^-PMA obtained above were dissolved in distilled water, respectively. The solutions were analyzed using HPLC (Agilent1200 LC, Santa Clara, CA, USA) for determination of the purity of the Ca^2+^-PMA. First, the Ca^2+^-PMA sample was separated on ZORBAXSB-C18 column (5.0 μm, 4.6 mm × 150 mm). The HPLC conditions were that mobile phase was 0.01 M (NH_4_)_2_HPO_4_ in 10.0% methanol solution which pH was adjusted to 2.7 using 1.0 M phosphoric acid and degassed using a microwave; flow rate was 1.0 mL/min; column temperature was 30°C; the sample volume was 20.0 μL; detector was waters 996 Diode-Array Detector; detection wavelength was 210 nm; sensitivity was 0.02 AUFS. The pure calcium malate and l-malic acid purchased from Sigma (St. Louis, MO, USA) were used as the standards.

### Measurement of the apparent molecular mass of the purified PMA

The apparent molecular weight of the purified PMA produced by the yeast strain MCW was evaluated by the Gel Permeation Chromatography (GPC) utilizing a Waters™ 1515 instrument combined with Multi-angle laser light scattering detector (MALLS) and concentration detector (RI) under the following conditions: Solvent: water; Flow rate: 0.500 mL/min; Laser wavelength: 658.0 nm; Calibration constant: 3.7138e-5 1/(V cm); Cell type: K5. The pullulan standards of varying known sizes (Showa, Denko) were used as the molecular mass references.
